# Supplementary material for: Predominant regional biophysical cooling from recent land cover changes in Europe
Source: Nat Commun. 2020 Feb 26;11:1066. doi: 10.1038/s41467-020-14890-0 (PMC7044322; doi:10.1038/s41467-020-14890-0)
Supplement: Supplementary file 1 — Supplementary Information [file 41467_2020_14890_MOESM1_ESM.pdf]

Predominant regional biophysical cooling from recent land cover changes in Europe

Supplementary Information

Bo Huang[a#], Xiangping Hu[a#], Geir-Arne Fuglstad[b], Xu Zhou[c], Wenwu Zhao[d], Francesco Cherubini[a*]

[a]*Industrial Ecology Programme, Department of Energy and Process Engineering, Norwegian University of Science and Technology (NTNU), Trondheim, Norway*

[b]*Department of Mathematical Sciences, Norwegian University of Science and Technology (NTNU), Trondheim, Norway*

[c]*TEL & CETES, Institute of Tibetan Plateau Research, Chinese Academy of Sciences, Beijing, China*

[d]*State Key Laboratory of Earth Surface Processes and Resource Ecology, Faculty of Geographical Science, Beijing Normal University (BNU), Beijing, China*

[#]*These authors equally contributed to this work.*

**Corresponding author: phone +47 73598942; email: francesco.cherubini@ntnu.no*

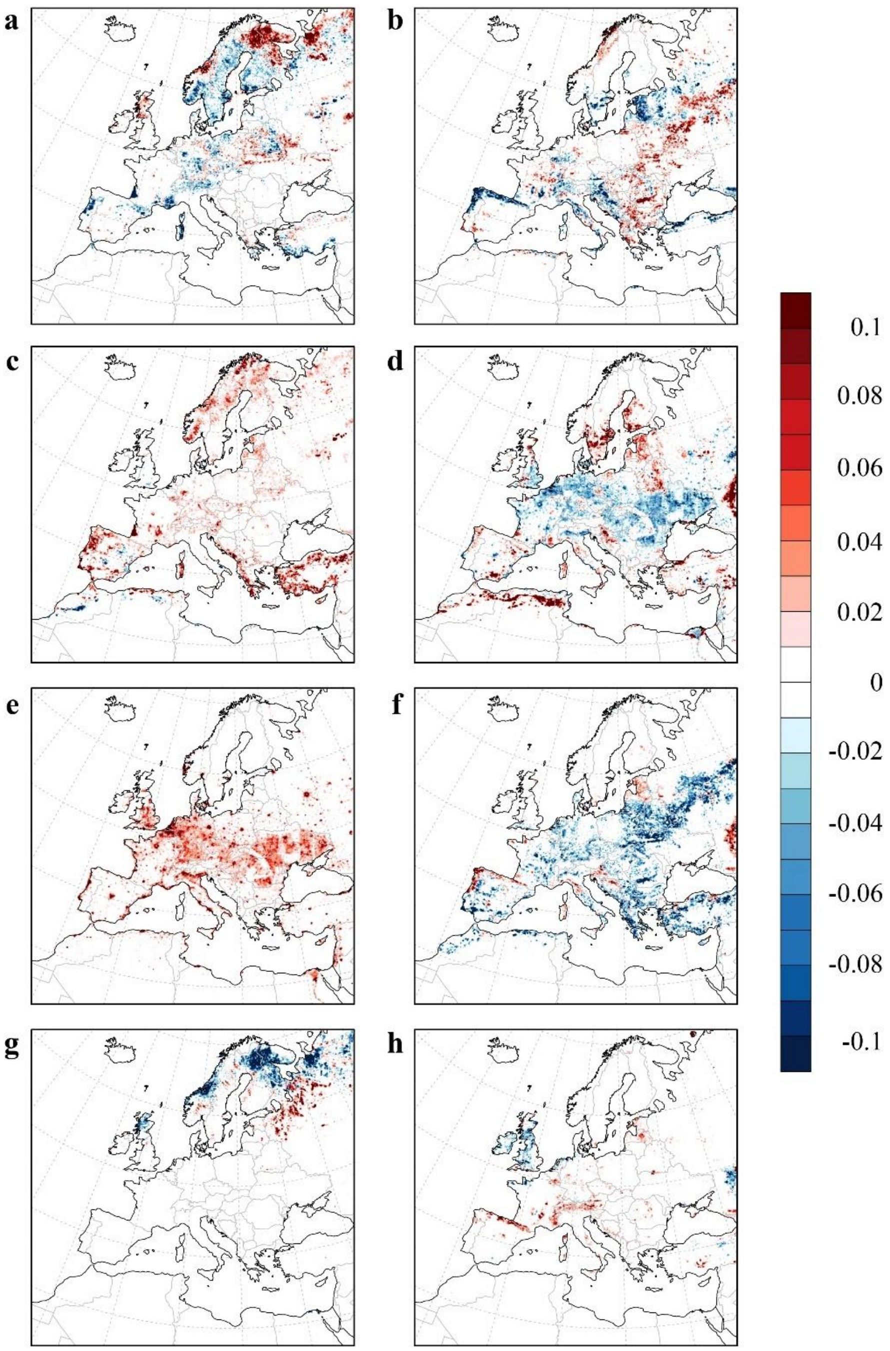

Supplementary Figure 1 Land cover transitions from 1992 to 2015 for the major IGBP land cover classes (LC2015-LC1992). The color bar indicates the fraction of each grid cell where the change occurred. Positive values mean expansion, negative values contraction. Legend: a) evergreen needleleaf forest, b) deciduous broadleaf forest, c) open shrubland, d) cropland, e) urban and built-up, f) cropland/natural vegetation mosaic, g) wetland, h) grassland.

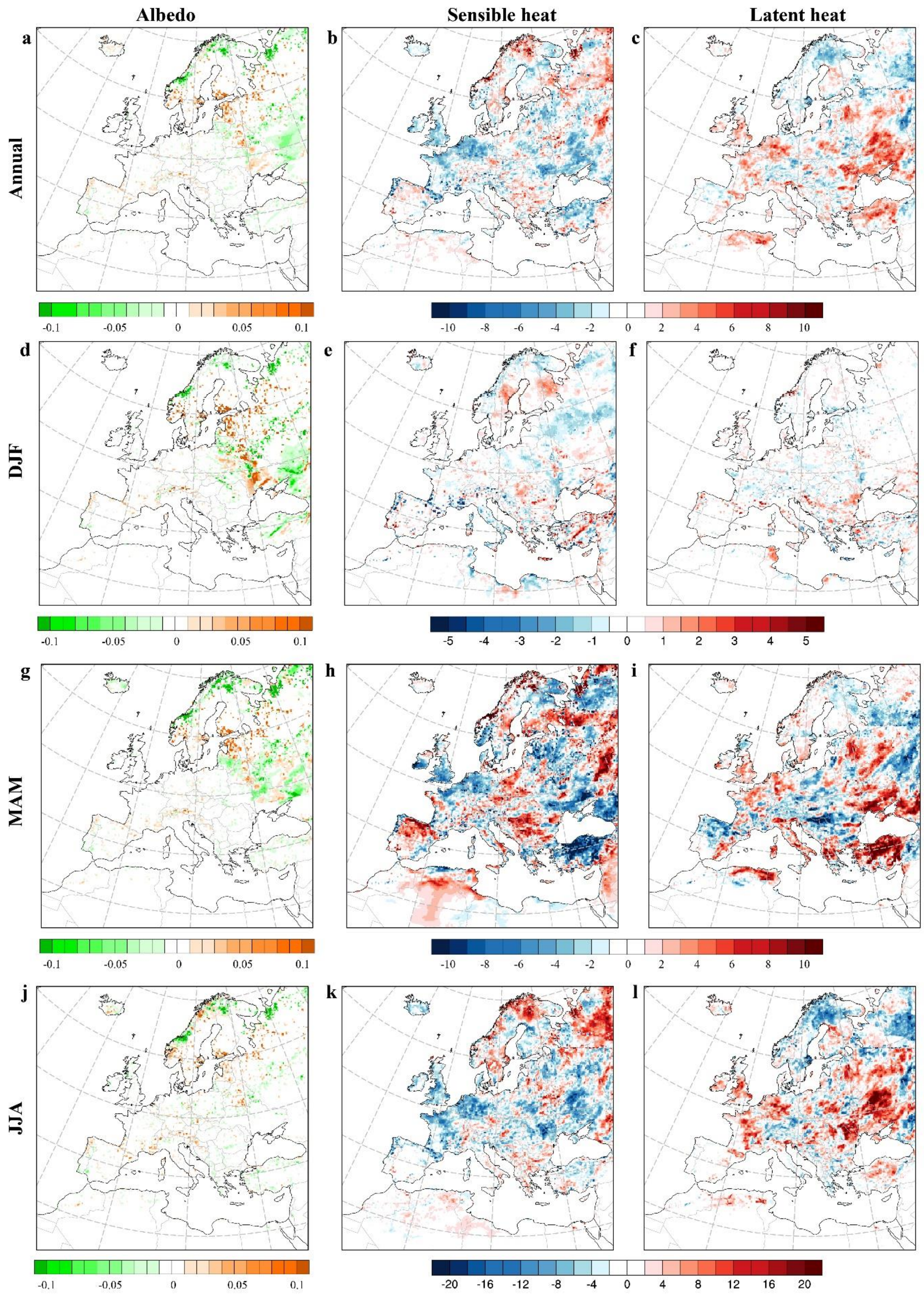

Supplementary Figure 2 Changes in surface albedo (a, d, g, j), sensible heat (b, e, h, k), and latent heat (c, f, i, l) in the annual mean (a, b, c), winter (d, e, f), spring (g, h, i), and summer (j, k, l) in the experiment "LC2015 – LC1992". Units of sensible and latent heat are W m^{-2}.

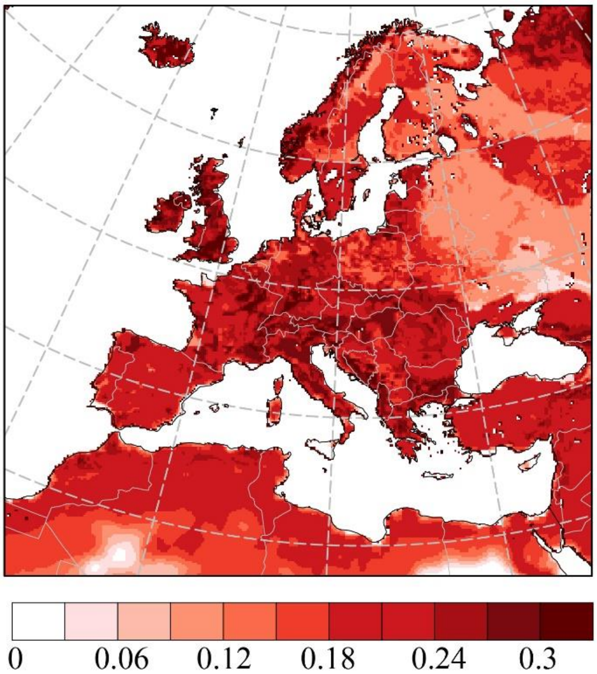

	Annual		Winter (DJF)		Summer (JJA)	
**Simulation**	**PCC**	**Bias (m^3 m^{-3})**	**PCC**	**Bias (m^3 m^{-3})**	**PCC**	**Bias (m^3 m^{-3})**
**a) LC1992**	0.95	-0.028	0.96	0.001	0.83	-0.062
**b) LC2015**	0.95	-0.029	0.96	-0.001	0.82	-0.063

Supplementary Figure 3 Annual mean values (LC1992) of total soil moisture in the simulation domain (m^3 m^{-3}). Similar values and trends are found for LC2015 (map not shown). The table reports the statistical scores of pattern correlation coefficients (PCC) and model bias of our soil moisture model outputs compared to the ESA CCI soil moisture observational database.

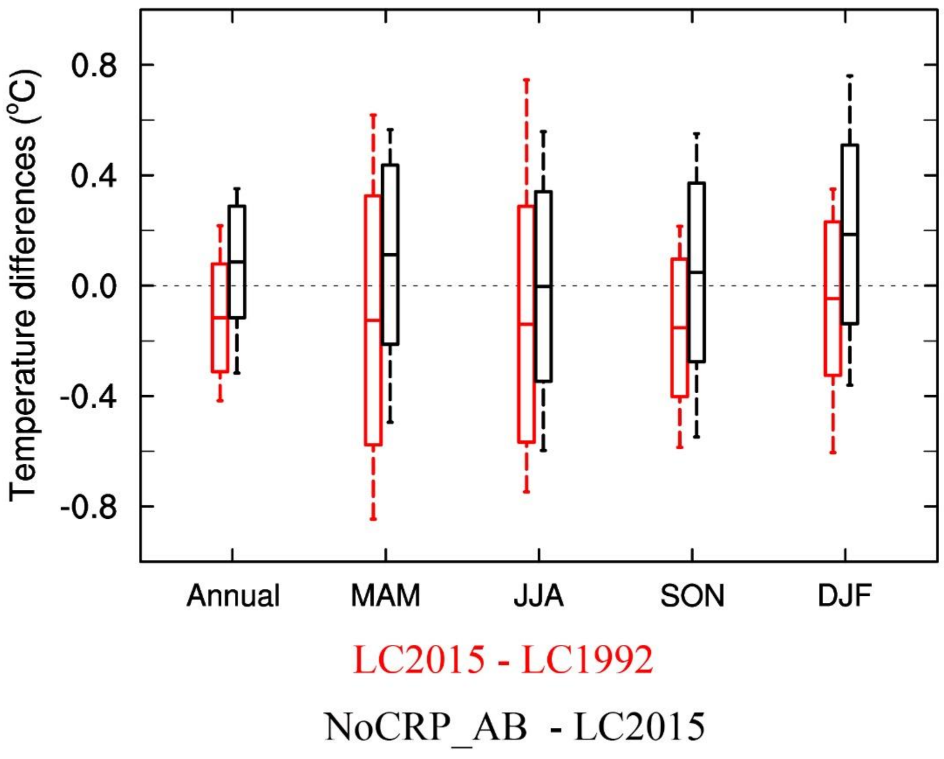

Supplementary Figure 4 Seasonality of the temperature differences caused by recent (1992-2015) land cover changes in Europe. Results shown are for both experiments “LC2015 – LC1992” and “NoCRP_AB – LC2015”. The boxes represent the range of one standard deviation, the lines in the middle of the boxes show the mean, and the whiskers the 5th and 95th percentiles. Results are shown for the annual average and the different seasons, spring (March, April, May, MAM), summer (June, July, August, JJA), autumn (September, October, November, SON), and winter (December, January, February, DJF).

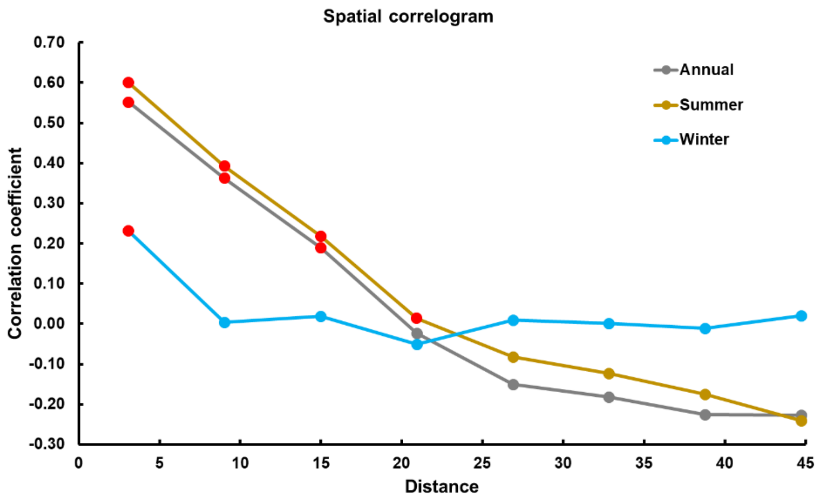

Supplementary Figure 5 Spatial correlogram plotting the autocorrelation coefficient *Moran's I* as a function of the distance class (i.e., number of grid cells) for annual average values and summer and winter seasonal means. The red dots indicate the statistically significant values ($p<0.05$).

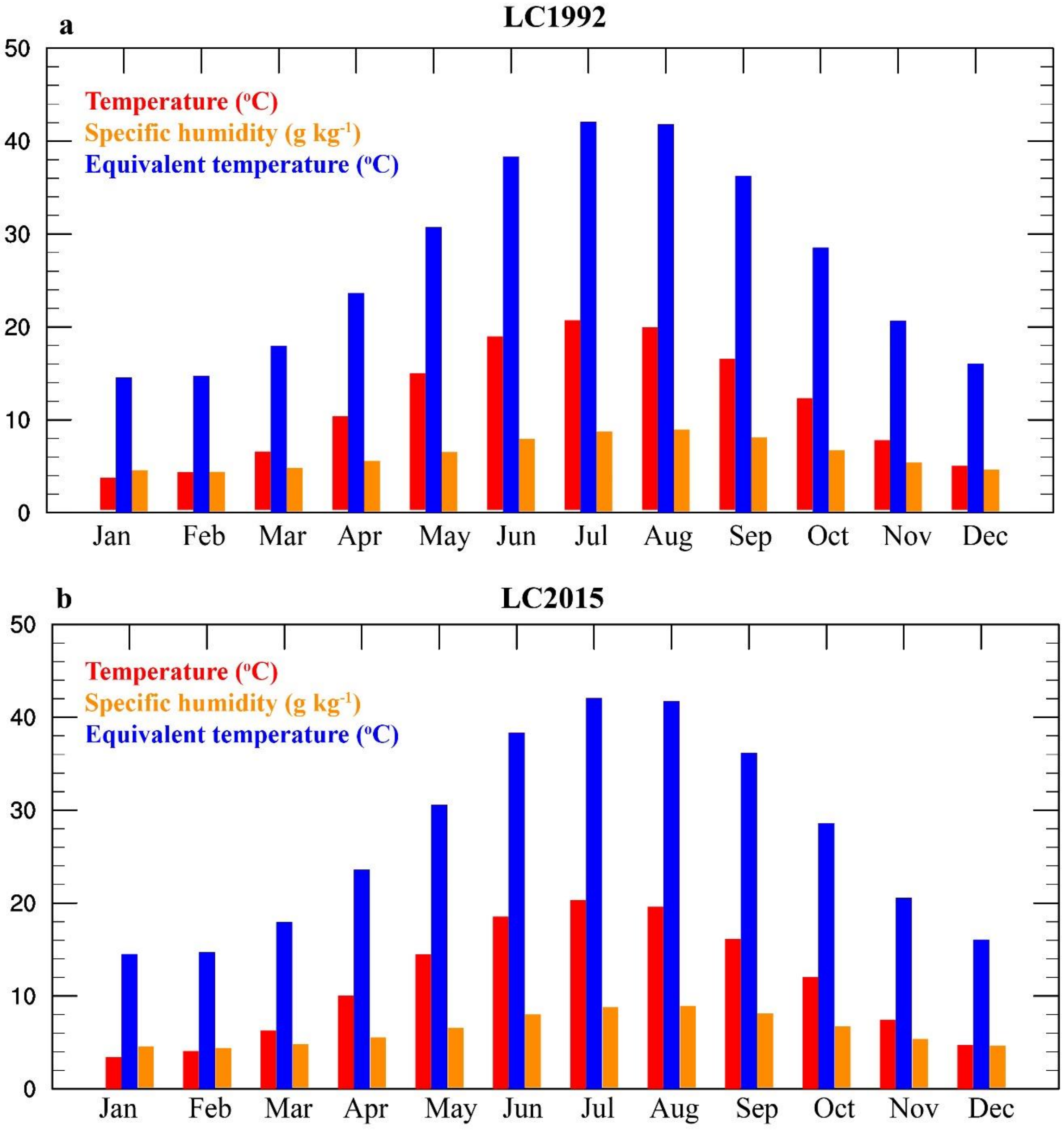

Supplementary Figure 6 Monthly mean climatology of temperature (*T*), specific humidity (*q*) and equivalent temperature (T_E) at 2 m for each individual simulation LC1992 and LC2015.

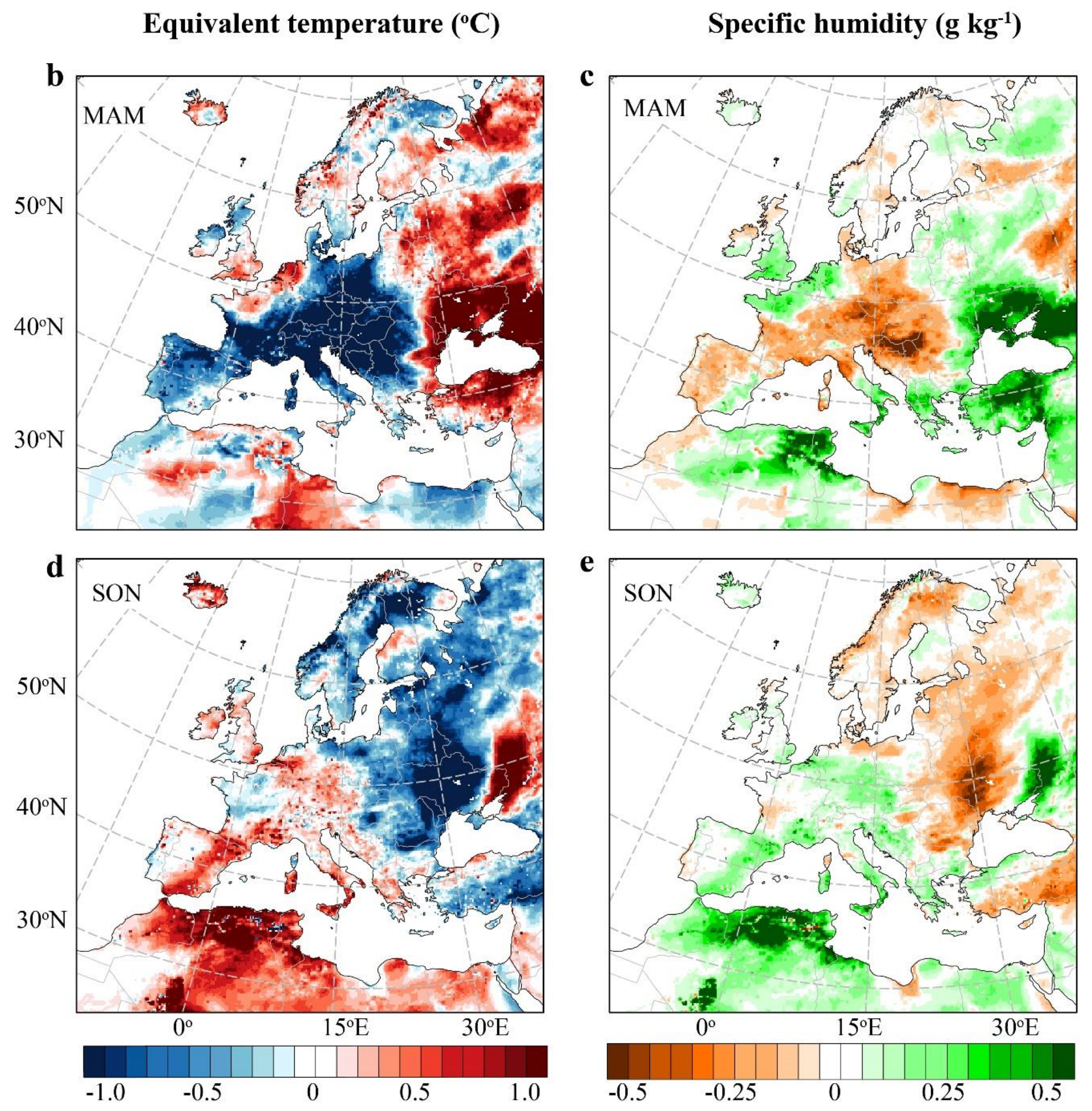

Supplementary Figure 7 Changes in T_E (b, d) and q (c, e) in spring (b, c) and autumn (d, e).

Supplementary Table 1 Comparison of the temperature effects for individual land cover transitions between our study and an observational dataset[1] (indicated as "Ref." in the table). Land cover classes refer to the IGBP classification system (see Figure 6 for explanation of the acronyms), and transitions are bi-directional. Transitions for which the estimated temperature change falls within the respective uncertainty ranges (2 standard errors, i.e. 95% confidence interval) are highlighted in green, those in yellow indicate the same sign in the temperature response, and those in red are mismatch. The white entries mean that either one or both datasets could not provide a confident estimate for the corresponding land transition. Note that there are differences on the metric and approaches used by the studies. The "Ref." dataset refers to the period 2008-2012, while for our study it is 1992 – 2015, and its temperature values are the mean (own calculation) between night and day land surface temperature observations, whereas our analysis focuses on air (2 m) temperature. Further, the reported temperature values in "Ref." refer to the area affected by the land use change only, while our study considers temperature values for grid cells where the LCC is only a fraction of the area of the cell. This can explain the generally lower values reported in our study.

Land Cover Transition	Europe		Subdomain A		Subdomain B	
	Our study	Ref.	Our study	Ref.	Our study	Ref.
ENF <=> OSL	0.19±0.04	0.20±0.08			0.22±0.12	0.18±0.12
ENF <=> GRA	0.23±0.12	0.11±0.06	0.16±0.12	0.24±0.06		
ENF <=> WET	0.02±0.04	-0.40±0.04			-0.05±0.06	-0.39±0.04
ENF <=> CRO	0.12±0.04	0.20±0.04	0.21±0.06	0.80±0.06	-0.14±0.10	-0.03±0.10
DBF <=> GRA	0.22±0.08	-0.16±0.14	0.18±0.10	-0.08±0.12	0.27±0.26	-0.29±0.10
DBF <=> WET					-0.12±0.08	-0.52±0.10
DBF <=> CRO	0.07±0.04	0.34±0.04	0.12±0.06	0.68±0.04	-0.10±0.10	-0.03±0.04
OSL <=> GRA	-0.11±0.12	0.10±0.14				
OSL <=> WET	-0.25±0.08	-0.18±0.10			-0.24±0.14	-0.17±0.10
OSL <=> CRO	-0.14±0.08	-0.27±0.06	-0.13±0.10	-0.12±0.14		
GRA <=> CRO	-0.04±0.06	0.43±0.04	0.04±0.08	0.67±0.04		

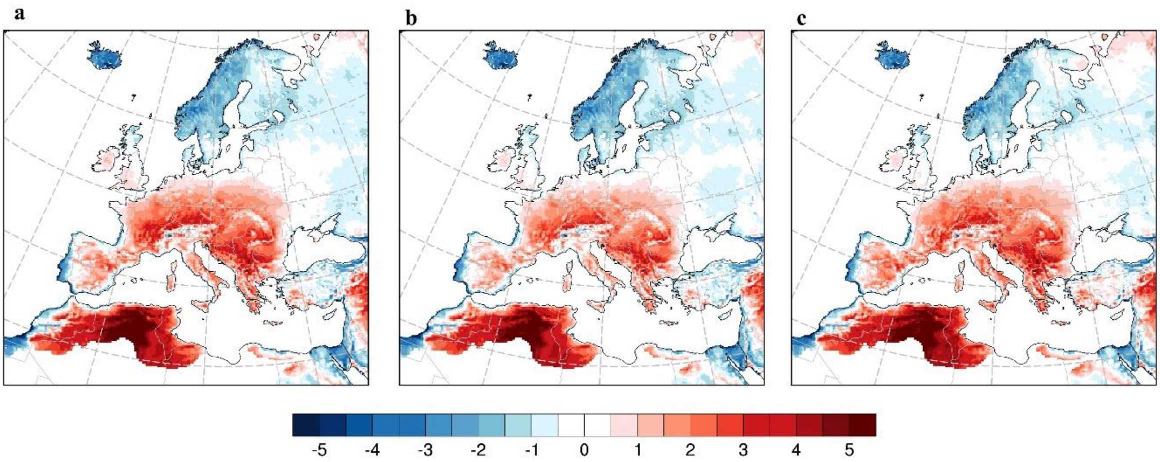

Simulation	PCC	Bias (°C)	RMSE (°C)
**a) LC1992 - EOBS**	0.98	0.35	1.87
**b) LC2015 - EOBS**	0.98	0.24	1.77
**c) IGBP - EOBS**	0.98	0.47	1.89

Supplementary Figure 8 Comparison of annual mean temperature difference between WRF model outputs using different land cover datasets and observations (EOBS), and respective performance statistics. a) WRF simulations with 1992 land cover class distribution from the CCI dataset; b) WRF simulations with 2015 land cover class distribution from the CCI dataset; c) WRF simulations with the default IGBP land cover dataset. Statistical scores refer to the bias, pattern correlation coefficient (PCC), and root mean square error (RMSE).

Supplementary Table 2 Comparison of monthly mean albedo values with standard deviation and pattern correlation coefficients (PCC) between our simulations and the observational dataset CLARA_A2. Similar values and trends are found for LC2015 (not shown).

	CLARA_A2		LC1992		
**Month**	**Mean**	**Standard deviation**	**PCC**	**Mean**	**Standard deviation**
1	0.23	0.18	0.91	0.25	0.12
2	0.32	0.24	0.92	0.27	0.12
3	0.31	0.21	0.91	0.25	0.14
4	0.24	0.14	0.90	0.20	0.13
5	0.19	0.08	0.95	0.17	0.08
6	0.18	0.04	0.97	0.17	0.05
7	0.18	0.03	0.98	0.17	0.05
8	0.18	0.03	0.97	0.16	0.04
9	0.17	0.04	0.97	0.16	0.04
10	0.19	0.06	0.94	0.15	0.05
11	0.20	0.10	0.84	0.15	0.06
12	0.18	0.06	0.89	0.17	0.08

Supplementary Table 3 Cross-walking table provided by the ESA-CCI to convert the UNLCCS classes to the standard IPCC classes[2]. This cross-walking table is only used for simplification and visualization purposes of the land cover changes shown in Figure 1 of the main paper.

	CCI-LC		IPCC classification									
			Agriculture	Forest	Grassland	Wetland	Settlement	Shrubland	Lichens and mosses	Sparse vegetation	Bare area	Water
	code	description										
1	0	no_data										
2	10	cropland_rainfed	X	-	-	-	-	-	-	-	-	-
3	11	cropland_rainfed_herbaceous_cover	X	-	-	-	-	-	-	-	-	-
4	12	cropland_rainfed_tree_or_shrub_cover	X	-	-	-	-	-	-	-	-	-
5	20	cropland_irrigated	X	-	-	-	-	-	-	-	-	-
6	30	mosaic_cropland	X	-	-	-	-	-	-	-	-	-
7	40	mosaic_natural_vegetation	X	-	-	-	-	-	-	-	-	-
8	50	tree_broadleaved_evergreen_closed_to_open	-	X	-	-	-	-	-	-	-	-
9	60	tree_broadleaved_deciduous_closed_to_open	-	X	-	-	-	-	-	-	-	-
10	61	tree_broadleaved_deciduous_closed	-	X	-	-	-	-	-	-	-	-
11	62	tree_broadleaved_deciduous_open	-	X	-	-	-	-	-	-	-	-
12	70	tree_needleleaved_evergreen_closed_to_open	-	X	-	-	-	-	-	-	-	-
13	71	tree_needleleaved_evergreen_closed	-	X	-	-	-	-	-	-	-	-
14	72	tree_needleleaved_evergreen_open	-	X	-	-	-	-	-	-	-	-
15	80	tree_needleleaved_deciduous_closed_to_open	-	X	-	-	-	-	-	-	-	-
16	81	tree_needleleaved_deciduous_closed	-	X	-	-	-	-	-	-	-	-
17	82	tree_needleleaved_deciduous_open	-	X	-	-	-	-	-	-	-	-
18	90	tree_mixed	-	X	-	-	-	-	-	-	-	-
19	100	mosaic_tree_and_shrub	-	X	-	-	-	-	-	-	-	-
20	110	mosaic_herbaceous	-	-	X	-	-	-	-	-	-	-
21	120	shrubland	-	-	-	-	-	X	-	-	-	-
22	121	shrubland_evergreen	-	-	-	-	-	X	-		-	-
23	122	shrubland_deciduous	-	-	-	-	-	X	-	-	-	-
24	130	grassland	-	-	X	-	-	-	-	-	-	-
25	140	lichens_and_mosses	-	-	-	-	-	-	X	-	-	-
26	150	sparse_vegetation	-	-	-	-	-	-	-	X	-	-
27	152	sparse_shrub	-	-	-	-	-	-	-	X	-	-
28	153	sparse_herbaceous	-	-	-	-	-	-	-	X	-	-
29	160	tree_cover_flooded_fresh_or_brakish_water	-	X	-	-	-	-	-	-	-	-
30	170	tree_cover_flooded_saline_water	-	X	-	-	-	-	-	-	-	-
31	180	shrub_or_herbaceous_cover_flooded	-	-	-	X	-	-	-	-	-	-
32	190	urban	-	-	-	-	X	-	-	-	-	-
33	200	bare_areas	-	-	-	-	-	-	-	-	X	-
34	201	bare_areas_consolidated	-	-	-	-	-	-	-	-	X	-
35	202	bare_areas_unconsolidated	-	-	-	-	-	-	-	-	X	-
36	210	water	-	-	-	-	-	-	-	-	-	X
37	220	snow_and_ice	-	-	-	-	-	-	-	-	-	-

Supplementary Table 4 Cross-walking table used to convert the CCI land cover classes to the IGBP land cover classes used as input to WRF (adapted from other references[1,3,4]).

		IGBP																				
	**CCI-LC**	1 Evergreen Needleleaf forest	2 Evergreen Broadleaf forest	3 Deciduous Needleleaf forest	4 Deciduous Broadleaf forest	5 Mixed forest	6 Closed shrubland	7 Open shrublands	8 Woody savannas	9 Savannas	10 Grasslands	11 Permanent wetlands	12 Croplands	13 Urban and built-up	14 Cropland/Natural vegetation mosaic	15 Snow and ice	16 Barren or sparsely vegetated	17 Water	18 Wooded Tundra	19 Mixed Tundra	20 Barren Tundra	21 Lake
0	no_data																					
10	cropland_rainfed	-	-	-	-	-	-	-	-	-	-	-	X	-		-	-	-	-	-	-	-
11	cropland_rainfed_herbaceous_cover	-	-	-	-	-	-	-	-	-	-	-	X	-		-	-	-	-	-	-	-
12	cropland_rainfed_tree_or_shrub_cover	-	-	-	-	-	-	-	-	-	-	-	X	-		-	-	-	-	-	-	-
20	cropland_irrigated	-	-	-	-	-	-	-	-	-	-	-	X	-		-	-	-	-	-	-	-
30	mosaic_cropland	-	-	-	-	-	-	-	-	-	-	-	-	-	X	-	-	-	-	-	-	-
40	mosaic_natural_vegetation	-	-	-	-	-	-	-	-	-	-	-	-	-	X	-	-	-	-	-	-	-
50	tree_broadleaved_evergreen_closed_to_open	-	X	-	-	-	-	-	-	-	-	-	-	-	-	-	-	-	-	-	-	-
60	tree_broadleaved_deciduous_closed_to_open	-	-	-	X	-	-	-	-	-	-	-	-	-	-	-	-	-	-	-	-	-
61	tree_broadleaved_deciduous_closed	-	-	-	X	-	-	-	-	-	-	-	-	-	-	-	-	-	-	-	-	-
62	tree_broadleaved_deciduous_open	-	-	-	X	-	-	-	-	-	-	-	-	-	-	-	-	-	-	-	-	-
70	tree_needleleaved_evergreen_closed_to_open	X	-	-	-	-	-	-	-	-	-	-	-	-	-	-	-	-	-	-	-	-
71	tree_needleleaved_evergreen_closed	X	-	-	-	-	-	-	-	-	-	-	-	-	-	-	-	-	-	-	-	-
72	tree_needleleaved_evergreen_open	X	-	-	-	-	-	-	-	-	-	-	-	-	-	-	-	-	-	-	-	-
80	tree_needleleaved_deciduous_closed_to_open	-	-	X	-	-	-	-	-	-	-	-	-	-	-	-	-	-	-	-	-	-
81	tree_needleleaved_deciduous_closed	-	-	X	-	-	-	-	-	-	-	-	-	-	-	-	-	-	-	-	-	-
82	tree_needleleaved_deciduous_open	-	-	X	-	-	-	-	-	-	-	-	-	-	-	-	-	-	-	-	-	-
90	tree_mixed	-	-	-	-	X	-		-	-	-	-	-	-	-	-	-	-	-	-	-	-
100	mosaic_tree_and_shrub	-	-	-	-	-	-	X	-	-	-	-	-	-	-	-	-	-	-	-	-	-
110	mosaic_herbaceous	-	-	-	-	-	-	X	-	-	-	-	-	-	-	-	-	-	-	-	-	-
120	shrubland	-	-	-	-	-	-	X	-	-	-	-	-	-	-	-	-	-	-	-	-	-
121	shrubland_evergreen	-	-	-	-	-	X	-	-	-	-	-	-	-	-	-	-	-	-	-	-	-
122	shrubland_deciduous	-	-	-	-	-	X	-	-	-	-	-	-	-	-	-	-	-	-	-	-	-
130	grassland	-	-	-	-	-	-	-	-	-	X	-	-	-	-	-	-	-	-	-	-	-
140	lichens_and_mosses	-	-	-	-	-	-	-	-	-	-	-	-	-	-	-	X	-	-	-	-	-
150	sparse_vegetation	-	-	-	-	-	-	-	-	-	-	-	-	-	-	-	X	-	-	-	-	-
152	sparse_shrub	-	-	-	-	-	-	-	-	-	-	-	-	-	-	-	X	-	-	-	-	-
153	sparse_herbaceous	-	-	-	-	-	-	-	-	-	-	-	-	-	-	-	X	-	-	-	-	-
160	tree_cover_flooded_fresh_or_brakish_water	-	-	-	-	-	-	-	-	-	-	X	-	-	-	-	-	-	-	-	-	-
170	tree_cover_flooded_saline_water	-	-	-	-	-	-	-	-	-	-	X	-	-	-	-	-	-	-	-	-	-
180	shrub_or_herbaceous_cover_flooded	-	-	-	-	-	-	-	-	-	-	X	-	-	-	-	-	-	-	-	-	-
190	urban	-	-	-	-	-	-	-	-	-	-	-	-	X	-	-	-	-	-	-	-	-
200	bare_areas	-	-	-	-	-	-	-	-	-	-	-	-	-	-	-	X	-	-	-	-	-
201	bare_areas_consolidated	-	-	-	-	-	-	-	-	-	-	-	-	-	-	-	X	-	-	-	-	-
202	bare_areas_unconsolidated	-	-	-	-	-	-	-	-	-	-	-	-	-	-	-	X	-	-	-	-	-
210	water	-	-	-	-	-	-	-	-	-	-	-	-	-	-	-	-	X	-	-	-	-
220	snow_and_ice	-	-	-	-	-	-	-	-	-	-	-	-	-	-	X	-	-	-	-	-	-

References

1 Duveiller, G., Hooker, J. & Cescatti, A. The mark of vegetation change on Earth's surface energy balance. *Nature Communications* **9**, 679, doi:10.1038/s41467-017-02810-8 (2018).

2 Defourny, P. *et al. Land Cover CCI: Product User Guide Version 2.0*, 2017).

3 Li, W. *et al.* Gross and net land cover changes in the main plant functional types derived from the annual ESA CCI land cover maps (1992–2015). *Earth Syst. Sci. Data* **10**, 219-234, doi:10.5194/essd-10-219-2018 (2018).

4 Poulter, B. *et al.* Plant functional type classification for earth system models: results from the European Space Agency's Land Cover Climate Change Initiative. *Geosci. Model Dev.* **8**, 2315-2328, doi:10.5194/gmd-8-2315-2015 (2015).
