# Supplementary material for: Predominant regional biophysical cooling from recent land cover changes in Europe
Source: Nat Commun. 2020 Feb 26;11:1066. doi: 10.1038/s41467-020-14890-0 (PMC7044322; doi:10.1038/s41467-020-14890-0)
Supplement: Supplementary file 3 — Description of Additional Supplementary Files [file 41467_2020_14890_MOESM3_ESM.pdf]

Description of Additional Supplementary Files

File Name: Supplementary Data 1
Description: The data and the code reproduce the results for the European domain using the ridge regression. Load the data in R and run the code.
