# Supplementary material for: Predominant regional biophysical cooling from recent land cover changes in Europe
Source: Nat Commun. 2020 Feb 26;11:1066. doi: 10.1038/s41467-020-14890-0 (PMC7044322; doi:10.1038/s41467-020-14890-0)
Supplement: Supplementary file 5 — Reporting Summary [file 41467_2020_14890_MOESM5_ESM.pdf]

nature research

Corresponding author(s): DBPR NCOMMS-19-05477A

Last updated by author(s): Jan 7 2020

Reporting Summary

Nature Research wishes to improve the reproducibility of the work that we publish. This form provides structure for consistency and transparency in reporting. For further information on Nature Research policies, see Authors & Referees and the Editorial Policy Checklist .

Statistics

For all statistical analyses, confirm that the following items are present in the figure legend, table legend, main text, or Methods section.

n/a	Confirmed	
☐	☒	The exact sample size (n) for each experimental group/condition, given as a discrete number and unit of measurement
☐	☒	A statement on whether measurements were taken from distinct samples or whether the same sample was measured repeatedly
☐	☒	The statistical test(s) used AND whether they are one- or two-sided<br>*Only common tests should be described solely by name; describe more complex techniques in the Methods section.*
☐	☒	A description of all covariates tested
☐	☒	A description of any assumptions or corrections, such as tests of normality and adjustment for multiple comparisons
☐	☒	A full description of the statistical parameters including central tendency (e.g. means) or other basic estimates (e.g. regression coefficient) AND variation (e.g. standard deviation) or associated estimates of uncertainty (e.g. confidence intervals)
☒	☐	For null hypothesis testing, the test statistic (e.g. F, t, r) with confidence intervals, effect sizes, degrees of freedom and P value noted<br>*Give P values as exact values whenever suitable.*
☒	☐	For Bayesian analysis, information on the choice of priors and Markov chain Monte Carlo settings
☒	☐	For hierarchical and complex designs, identification of the appropriate level for tests and full reporting of outcomes
☒	☐	Estimates of effect sizes (e.g. Cohen's d, Pearson's r), indicating how they were calculated

Our web collection on statistics for biologists contains articles on many of the points above.

Software and code

Policy information about availability of computer code

Data collection	As explained in the method, we used different datasets of land cover changes and climate. The main post-processed data are available as supplementary data
Data analysis	We provide a code (in R) used in the statistical analysis as supplementary data

For manuscripts utilizing custom algorithms or software that are central to the research but not yet described in published literature, software must be made available to editors/reviewers. We strongly encourage code deposition in a community repository (e.g. GitHub). See the Nature Research guidelines for submitting code & software for further information.

Data

Policy information about availability of data

All manuscripts must include a data availability statement. This statement should provide the following information, where applicable:

- Accession codes, unique identifiers, or web links for publicly available datasets
- A list of figures that have associated raw data
- A description of any restrictions on data availability

Data availability

A post-processed dataset of the temperature changes in the European domain is available as Supplementary Data file. Additional data are available from the corresponding author upon reasonable request.

Field-specific reporting

Please select the one below that is the best fit for your research. If you are not sure, read the appropriate sections before making your selection.

☐ Life sciences ☐ Behavioural & social sciences ☒ Ecological, evolutionary & environmental sciences

For a reference copy of the document with all sections, see nature.com/documents/nr-reporting-summary-flat.pdf

Ecological, evolutionary & environmental sciences study design

All studies must disclose on these points even when the disclosure is negative.

Study description	we use the high resolution data in combination with a regional climate model (the Weather Research and Forecasting model, WRF) to quantify the effects that land cover differences have had on European climate between 1992 and 2015.
Research sample	The European Space Agency (ESA) Climate Change Initiative (CCI) land cover (LC) product is used to map changes in vegetation cover.<br><br>The initial and lateral boundary conditions for the regional climate model simulations are from the European Centre for Medium-Range Weather Forecasts Interim reanalysis (ERA-Interim)
Sampling strategy	n.a.
Data collection	n.a.
Timing and spatial scale	n.a.
Data exclusions	n.a.
Reproducibility	We used open source dataset and models, and specify in the methods the parameterization. We provide as supplementary data the code used for the statistical analysis
Randomization	n.a.
Blinding	n.a.

Did the study involve field work? ☐ Yes ☒ No

Reporting for specific materials, systems and methods

We require information from authors about some types of materials, experimental systems and methods used in many studies. Here, indicate whether each material, system or method listed is relevant to your study. If you are not sure if a list item applies to your research, read the appropriate section before selecting a response.

Materials & experimental systems

n/a	Involved in the study	
☒	☐	Antibodies
☒	☐	Eukaryotic cell lines
☒	☐	Palaeontology
☒	☐	Animals and other organisms
☒	☐	Human research participants
☒	☐	Clinical data

Methods

n/a	Involved in the study	
☒	☐	ChIP-seq
☒	☐	Flow cytometry
☒	☐	MRI-based neuroimaging
